# Static magnetic field enhances the anticancer efficacy of capsaicin on HepG2 cells via capsaicin receptor TRPV1

**DOI:** 10.1371/journal.pone.0191078

**Published:** 2018-01-16

**Authors:** Wei-Ting Chen, Guan-Bo Lin, Shu-Hui Lin, Chueh-Hsuan Lu, Chih-Hsiung Hsieh, Bo-Lun Ma, Chih-Yu Chao

**Affiliations:** 1 Department of Physics, Lab for Medical Physics & Biomedical Engineering, National Taiwan University, Taipei, Taiwan; 2 Biomedical & Molecular Imaging Center, National Taiwan University College of Medicine, Taipei, Taiwan; 3 Institute of Applied Physics, National Taiwan University, Taipei, Taiwan; Sudbury Regional Hospital, CANADA

## Abstract

Static magnetic field (SMF) has shown some possibilities for cancer therapies. In particular, the combinational effect between SMF and anti-cancer drugs has drawn scientists’ attentions in recent years. However, the underlying mechanism for the drug-specific synergistic effect is far from being understood. Besides, the drugs used are all conventional chemotherapy drugs, which may cause unpleasant side effects. In this study, our results demonstrate for the first time that SMF could enhance the anti-cancer effect of natural compound, capsaicin, on HepG2 cancer cells through the mitochondria-dependent apoptosis pathway. We found that the synergistic effect could be due to that SMF increased the binding efficiency of capsaicin for the TRPV1 channel. These findings may provide a support to develop an application of SMF for cancer therapy. The present study offers the first trial in combining SMF with natural compound on anti-cancer treatment, which provides additional insight into the interaction between SMF and anti-cancer drugs and opens the door for the development of new strategies in fighting cancer with minimum cytotoxicity and side effects.

## Introduction

Magnetic fields with different intensities naturally exist in the world. The Earth’s magnetic field of around 0.3–0.6 Gauss is required for many animals to navigate during migration. A permanent magnet composed of many magnetic dipoles produces its own static magnetic field (SMF). SMFs are time-independent fields and can interact directly with moving charges. To date, there were many in vivo and in vitro studies highlighting the beneficial effects on biological systems with SMF exposure [1]. For example, Jing *et al*. showed that 180 mT SMF presented a beneficial effect on diabetic wound healing in 3-month-old rats [2]. The acceleration of new bone tissue formation [3], the stimulation of neurite outgrowth [4, 5], the pain relief [1, 6], blood pressure modulation [7], and the anti-inflammatory effects [8] by SMF exposure were also observed. One fascinating therapeutic use of SMF is the cancer-fighting application, in which researches have shown that solo SMF has the potential to inhibit cancer cell through different mechanisms, such as retarded vessel maturation by anti-angiogenesis [9], reduction in immunoreactive p53 expression [10], and influence on the calcium signaling pathway [11].

Although the above results provided some understandings about the effect of SMF on cancer cells, most studies claimed that SMF as monotherapy is not sufficiently successful, but prefers the combinational effects with chemotherapy drugs [10, 12]. For example, the efficacy of conventional chemotherapy drugs such as Taxol [13, 14], Cisplatin [15], and Doxorubicin [12] were enhanced by moderate SMF with different intensities. These data indicate that SMF might be an assisted method for cancer chemotherapy, allowing minimum dosage of drugs to produce maximum curative effect. However, the mechanism of the synergistic effect between SMF and anticancer drugs is far from being understood. Some researches provided evidence of SMF-induced changes of cell surface structure increasing the permeability of cell membranes [12]. Gellrich *et al*. found SMF increased tumor microvessel leakiness and improved antitumoral efficacy in combination with paclitaxel in vivo [14]. Nevertheless, the increased permeability can hardly explain the drug-specific synergism or antagonism behavior between SMF and different drugs [12, 13, 16], and so far little attention has been put on the interaction between SMF and drugs. Besides, the drugs used in these experiments are all conventional chemotherapy drugs, which may cause unpleasant side effects. Nowadays, there has been an emerging area of cancer prevention and cure focused on natural compounds, especially the dietary products because of their low toxicity and potent efficacy. In this work, we choose capsaicin to be studied, which is the major pungent ingredient of the hot chili peppers and responsible for their spicy flavor. In particular, recent studies have demonstrated the anti-cancer activity of capsaicin in various types of cancer models [17, 18]. Capsaicin binds to distinct cell surface receptors, including the most well-known ion channel transient receptor potential vanilloid 1 (TRPV1) [19]. TRPV1 channel is a non-selective cation channel, which has an important role in heat sensors. This ion channel is activated by several physical, chemical and biological factors [20]. Drugs which could regulate TRPV1 channel activity could be useful for the treatment of conditions ranging from chronic pain to hearing loss [21]. Previous studies have shown that TRPV1 acts as a tumor suppressor through inducing tumor apoptosis upon activation by its agonist [22]. Therefore, in this study, the involvement of TRPV1 was also investigated in the HepG2 cells undergoing capsaicin and SMF induced apoptosis.

Previous research had shown the time-dependent modifications of HepG2 cells during exposure to SMF [11]. However, the data indicated that the biological effects of 6 mT SMF on HepG2 cells are not cytotoxic. Besides, capsaicin-induced apoptosis was also observed in HepG2 cells [23]. Therefore, the objective of this study was to investigate the synergistic anti-cancer effect of SMF and capsaicin on HepG2 cells.

In this paper, we study the combinational anti-cancer effects of SMF with capsaicin by means of the cell viability as well as light microscopy. The apoptotic signaling pathways were evaluated by Western blot analysis and immunofluorescence microscopy. Our results demonstrated that SMF enhanced the effect of natural compound, capsaicin, to inhibit the growth of cancer cells for the first time. The TRPV1 activation was confirmed to participate in the synergistic anti-cancer effect by the antagonist experiment and the change of calcium in cytoplasm related to the treatments. These data first indicate that combining SMF exposure with capsaicin is a promising therapy strategy, which sheds light on new anti-cancer treatments in combination of magnetic field and other nature compounds.

## Materials and methods

### Cell culture and treatment

**Fig 1:**
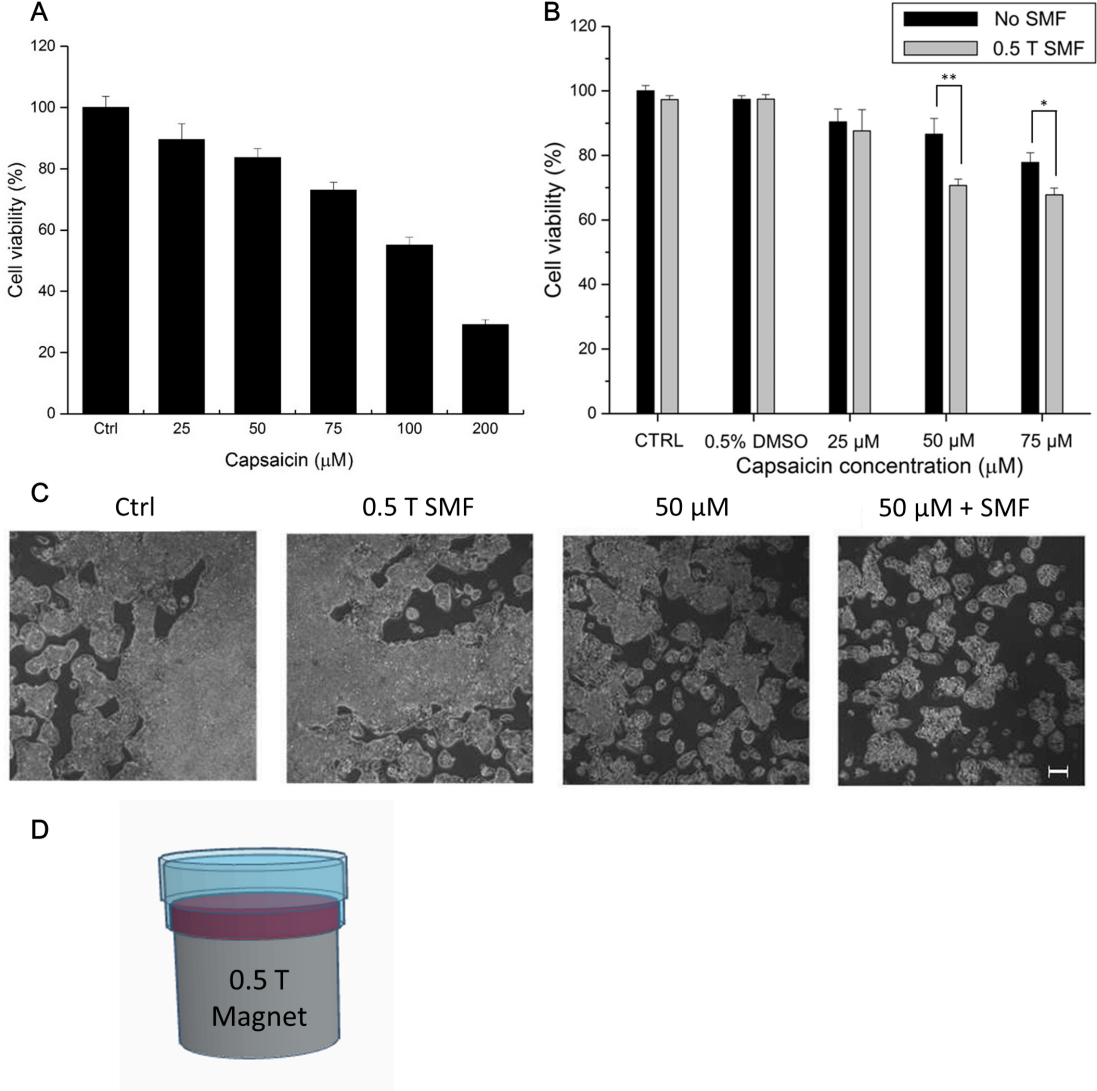
Viability and light microscopy images of HepG2 cells. (A) Dose-response curve of HepG2 cells treated with different concentrations of capsaicin for 72 h. (B) Cells were treated with different concentrations of capsaicin with or without 0.5 T SMF for 72 h and then the residual cell viability was measured by MTT assay. (C) Representative bright field images after treatment for 72 h. (D) SMF exposure setup. Data represent the mean ± SD. *p < 0.05 and **p < 0.01. Scale bar: 300 μm.

The HepG2 cell was purchased from Bioresource Collection and Research Center and cultured in DMEM medium (Hyclone) supplemented with 5% fetal bovine serum, 100 units/ml penicillin, and 100 μg/ml streptomycin. Cells were maintained in a humidified atmosphere composed of 5% CO_2_ and 95% air at 37°C. Cells were plated in 3 cm culture dishes at 1 x 10^5^ cells per dish 24 h before they were treated with or without capsaicin (Sigma) and SMFs for 72 h. The SMF groups were placed on cylindrical permanent magnets (Fig 1D) derived from the rare earth material neodymium iron boron. The magnetic flux density of ~ 0.5 T was measured in the center of the cylindrical magnet by means of a digital gaussmeter (Model: TM-701; Kanetec Co., Japan). TRPV1 antagonist SB-705498 (Targetmol) was incubated with the cells 30 min before SMF and capsaicin treatments.

### Cell viability assay

Viability of HepG2 cells after treatments with SMF and capsaicin was assessed by MTT assay. Briefly, the medium was replaced with MTT solution (0.5 mg/mL in DMEM) and incubated at 37°C for 3.5 h. During this period, the ability of mitochondrial dehydrogenases to reduce the MTT to formazan is an indicator of cellular viability. The supernatants were then replaced with DMSO and incubated for complete solubility of formazan crystal. The optical density of each well was then determined at 570 nm using a FLUOstar OPTIMA microplate reader. The cell viability was calculated based on the intensity of formazan, and expressed as percentage of non-treated control.

### Western blot analysis

Protein expression levels were investigated by Western blot of whole HepG2 cell lysates. Adherent cells were washed with cold PBS, and scraped off from culture dishes in RIPA lysis buffer supplemented with protease and phosphatase inhibitor cocktail. The collected cells were lysed by incubation with the lysis buffer for 30 min on ice. Lysates were vortexed and centrifuged at 15,000 rpm for 30 min at 4°C. After centrifugation, the supernatants were collected and the protein concentrations were determined by Bradford Protein Assay (BioShop, Canada) using bovine serum albumin as standard. Each lysate was denatured for 5 min at 95°C in SDS-polyacrylamide gel electrophoresis (SDS-PAGE) 5x sample buffer. An equal amount of proteins (30 μg) were resolved by 10% SDS-PAGE at 40 mA for 2 h and transferred to a polyvinylidene fluoride (PVDF) membrane. The PVDF membrane was stained with Ponceau S to verify the proper transfer. Nonspecific protein binding was blocked in 5% nonfat dry milk in TBST (20 mM Tris-base, pH 7.6; 0.15 M NaCl, 0.1% Tween 20) for 1 h at RT. After blockage, the membranes were probed with specific primary antibodies diluted in blocking solution overnight at 4°C. The membranes were washed four times with TBST solution for 5 min each, and then incubated with horseradish peroxidase-conjugated goat anti-rabbit secondary antibodies (1:500 dilution; Jackson ImmunoResearch Inc.) in blocking solution. The density of the targeted bands was visualized by the enhanced chemoluminescence (ECL) substrate (Advansta). Signals were detected by image system (Amersham Imager 600; GE Healthcare Life Science).

### Immunofluorescence microscopy

At the end of capsaicin and SMF treatments, cells were washed with PBS and fixed with 3.7% paraformaldehyde (PFA; Sigma) in PBS for 15 min at RT. Fixed cells were then permeabilized with 0.1% Triton X-100 in PBS for 15 min. Nonspecific protein binding was blocked with 1% BSA in PBS for 20 min at RT. After washing three times with PBS, the cells were incubated with anti-β-tubulin (1:1000 dilution; Abcam) and anti-active caspase-3 (1:800 dilution; Cell Signaling Technology) primary antibodies overnight at 4°C. The cells were then washed three times in PBS, and incubated in the Alexa Fluor 488-conjugated donkey anti-goat secondary antibody and the Alexa Fluor 647-conjugated donkey anti-rabbit secondary antibody (1:500 dilution; Jackson ImmunoResearch Inc.) for 1 h at 37°C in the dark. The coverslips were mounted to slides in mounting medium with DAPI (Abcam). The mounted samples were examined by a Zeiss LSM 880 inverted laser scanning confocal microscope. Pictures of randomly selected areas were taken for each sample and representative micrographs are shown in the figures.

### Flow cytometry

Apoptosis was analysed by flow cytometry with the Annexin V-FITC and propidium iodide (PI) double-staining method (BD Biosciences). Cells used for flow cytometry were collected by trypsinization and washed three times with ice-cold PBS before they were resuspended in 1× binding buffer. Then, 100 μL of the solution (2 x 10^5^ cells) was transferred to a 5 mL culture tube. Cells were stained with Annexin V and PI for 15 min at 25°C in the dark. Another 400 μL of the 1× binding buffer was added to each tube before they were analysed by flow cytometry.

### Intracellular calcium assay

The levels of calcium in HepG2 cells was determined by Rhod-4 no wash calcium assay kit (Abcam). Approximately 4×10^4^ cells/well of HepG2 cells were seeded in 96-well black wall clear bottom plates overnight. The SMF was treated 4 h before the experiment. The growth medium was replaced with HHBS medium (Gibco) and 100 μL/well of Rhod-4 dye-loading solution was added into the cell plates 1 h before the experiment. The calcium concentration was measured by monitoring the fluorescence intensity at Ex/Em = 532/555 nm using the FlexStation 3 microplate reader (Molecular Devices) just after the capsaicin administration.

### Statistical analysis

Each data point represents the average from three independent experiments (n = 3) and is expressed as mean ± standard deviation. Differences of statistical significance were determined by a one-way analysis of variance (ANOVA), followed by Tukey’s post-hoc test. *p < 0.05 and **p < 0.01 compared with control. Analyses were carried out using OriginPro graphing software (OriginLab).

## Results

### 0.5 T SMF increases the inhibition effect of capsaicin on HepG2 cell proliferation

We used MTT assay to examine the viability of HepG2 cell under SMF and/or capsaicin administration. Cells were treated with capsaicin, combined with or without SMF exposure for 72 h. Fig 1A shows that the HepG2 cells were inhibited by capsaicin in a dose-dependent manner. Cell proliferation was reduced significantly when the concentration of capsaicin was above 100 μM. Previous studies had shown that 100 μM capsaicin induced significant neurotoxicity in cultured neuron cells [24]. In this study, we aim to investigate the combinational effect of capsaicin and SMF, so we choose the concentrations of capsaicin below 100 μM. We first evaluate the effect of 0.5 T SMF on the viability of HepG2 cells. Our results show that the HepG2 cell proliferation of the SMF treated group did not differ from that of the control group, indicating that SMF alone will not cause inhibition effect on HepG2 cells. Interestingly, the experiments showed that 0.5 T SMF could increase the efficacy of capsaicin in HepG2 cells. For example, the viability of HepG2 cells in the group treated with 50 μM capsaicin can be further reduced by about 20% in combination with 0.5 T SMF (Fig 1B). For 75 μM capsaicin, which already reduced the viability of HepG2 cells, its inhibition effect was also increased by 0.5 T SMF. Light microscopy images also showed significant inhibiting effect in the SMF + capsaicin group after 72 h treatment (Fig 1C).

### Effect of capsaicin and SMF on expression of Bax and Bcl-2 proteins

**Fig 2:**
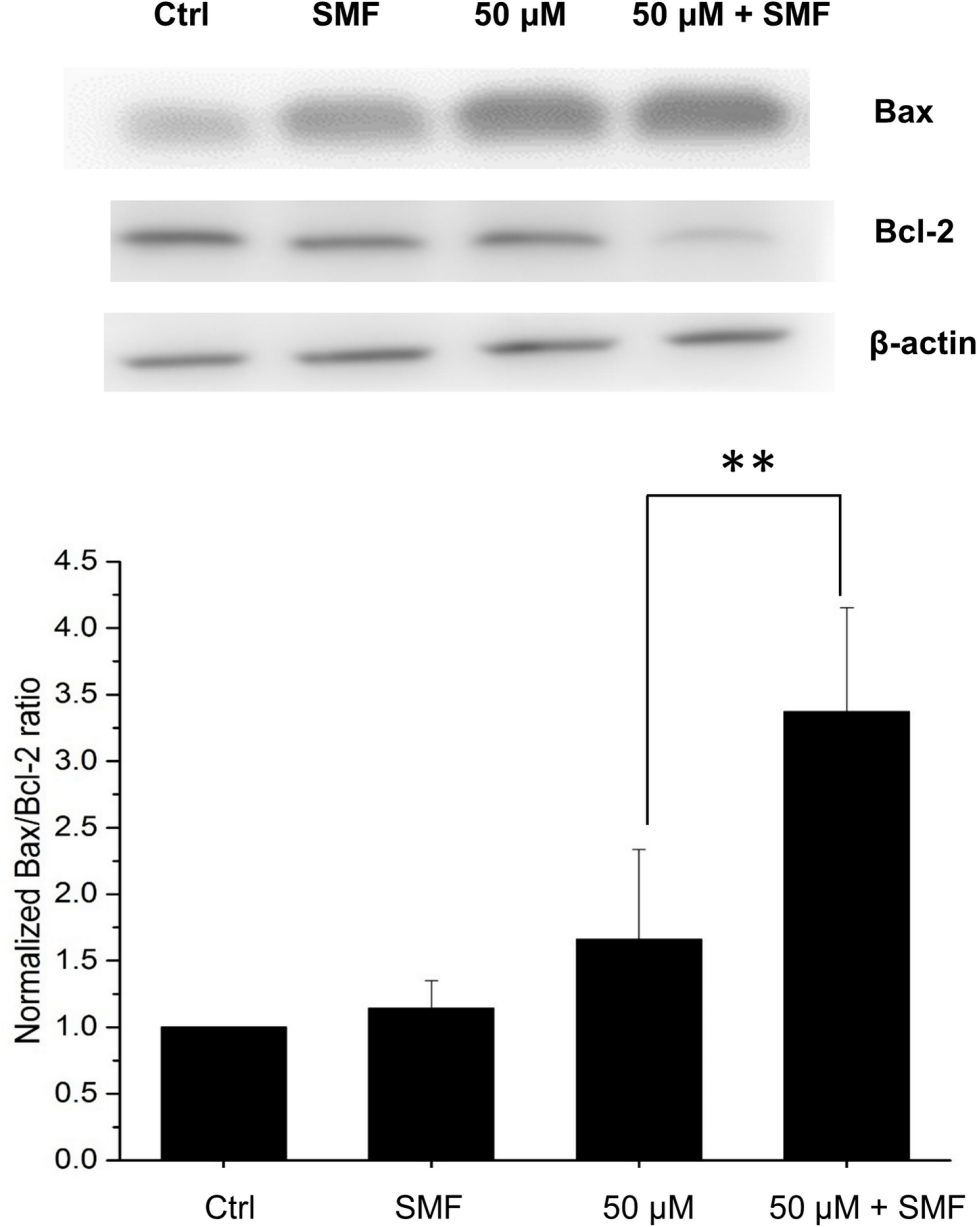
Western blot analysis of Bax and Bcl-2 proteins. Immunoblot results are from a representative experiment with β-actin as loading control. The density of Bax/Bcl-2 ratio was converted to grayscale value and normalized to the control. Data represent the mean ± SD. **p < 0.01.

To investigate the role of proteins involved in capsaicin and SMF-induced cell death, we carried out Western blotting of Bcl-2 family proteins Bax and Bcl-2. It was known that the increased Bax/Bcl-2 ratio indicates the up-regulation of apoptotic signaling pathways [25–27]. The Western blot result (Fig 2) shows that 50 μM capsaicin caused an elevation in Bax protein level, and the level was further enhanced under SMF stimulation. On the contrary, the Bcl-2 protein level was decreased in the capsaicin and capsaicin plus SMF treated groups. As a result, the increased Bax/Bcl-2 ratio in the capsaicin treated group was further elevated under SMF stimulation. The result confirmed that the potency of capsaicin was indeed increased under 0.5 T SMF exposure.

### SMF enhances the effect of capsaicin-induced apoptosis

**Fig 3:**
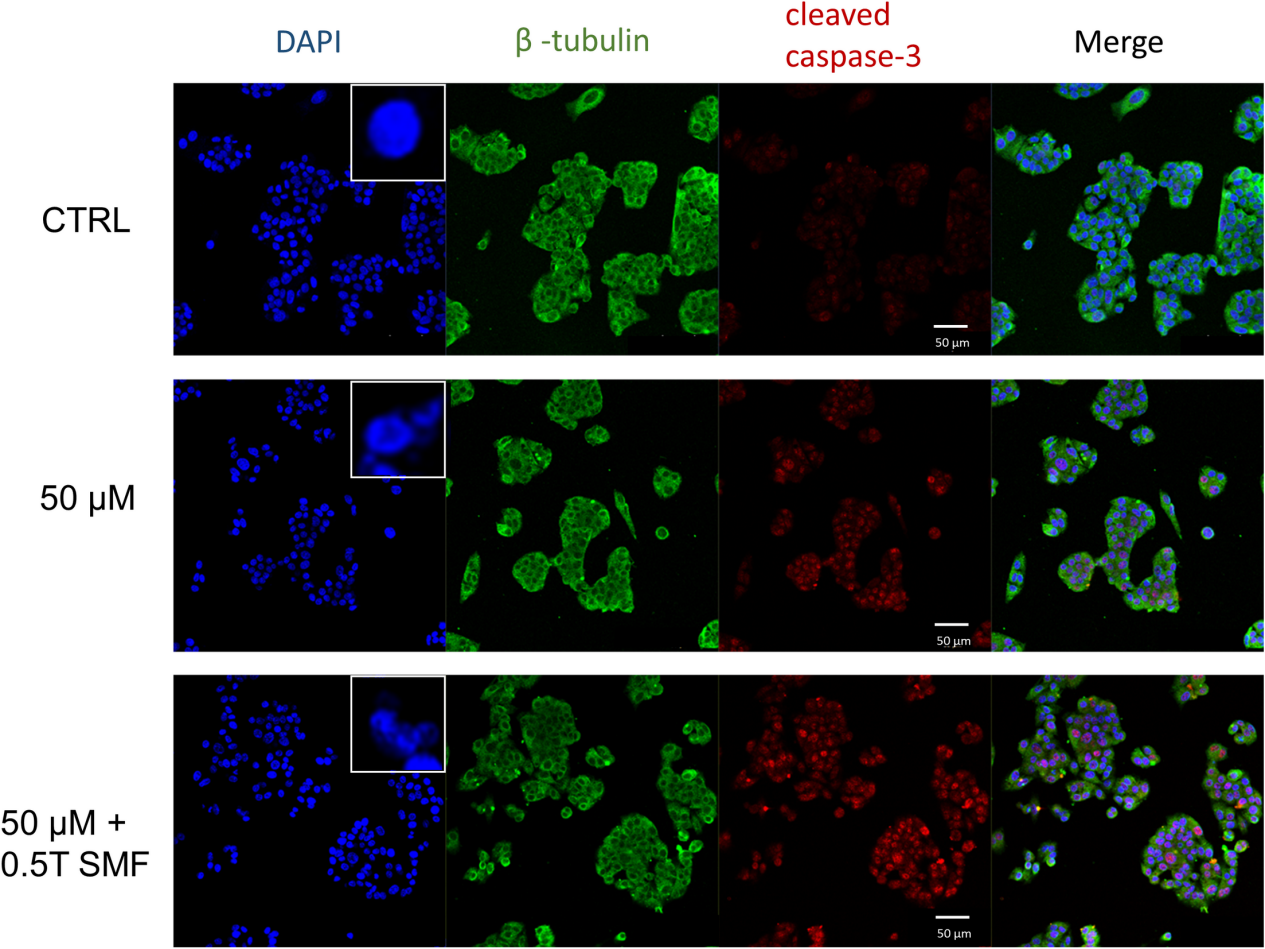
SMF exposure aggravated active caspase-3 intensities. Confocal images of HepG2 cells treated with 50 μM capsaicin with or without SMF for 72 h. Red fluorescence represents active caspase-3 staining; cell shapes were marked with β-tubulin green fluorescence and nuclei were labeled with DAPI. The upper right corner in the DAPI group showed the zoomed-in view of nuclei. Scale bar: 50 μm.

To further understand the mechanism of capsaicin and SMF-induced cell death, we investigated the cleaved caspase-3 expression on HepG2 cells by confocal microscope. Caspase-3 is one of the key executioners of apoptotic events in cells, and since it serves as a convergent downstream from different signaling pathways, it is well suited as an indicator in an apoptosis assay. In experiments, HepG2 cells were double-stained with β-tubulin and active caspase-3, and DAPI was used to visualize the nuclei of cells. As seen in Fig 3, the cell shape was clearly outlined by β-tubulin marker. Typical morphological changes of cell apoptosis, such as blebbing and nucleus fragmentation can be detected in DAPI staining (zoomed-in views in Fig 3). An antibody against cleaved caspase-3 showed negligible signal in the control cells, whereas the active caspase-3 signals were increased in capsaicin treated cells, and further increased under SMF treatment (Fig 3). The cleaved caspase-3 immunofluorescent signals caused by 50 μM capsaicin treatment were significantly increased under SMF exposure, which again demonstrated the synergistic effect of SMF and capsaicin.

### SMF increases capsaicin-induced HepG2 cell apoptosis

**Fig 4:**
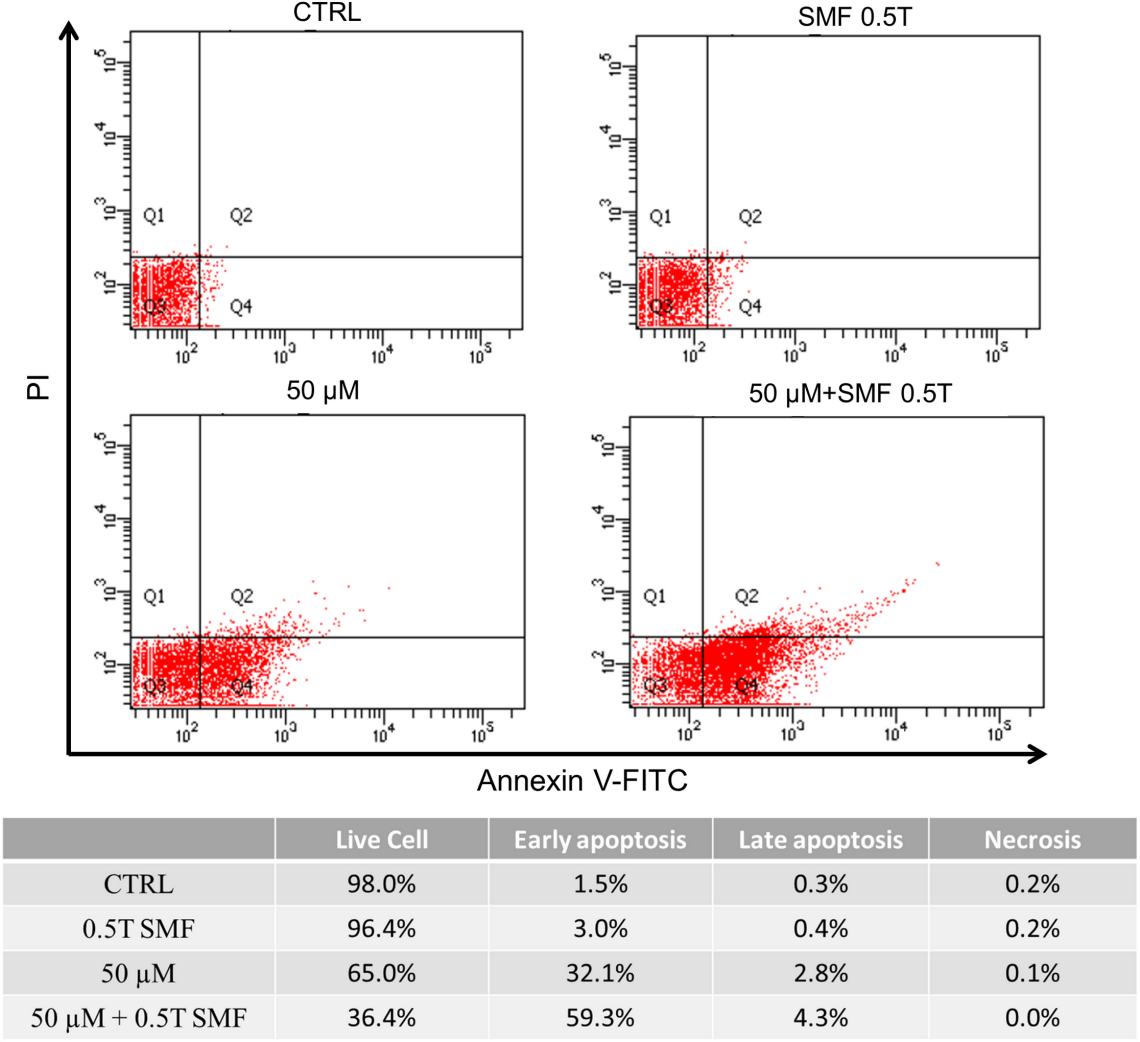
In vitro assessment of apoptosis via Annexin V-FITC/PI double staining. Flow cytometric analysis of Annexin V-FITC and PI-staining. The cells in the lower right quadrant indicate Annexin V-positive, PI-negative, early apoptotic cells. Percentages of viable, early and late apoptotic cells in different conditions were listed in the table below.

To confirm whether SMF and capsaicin decreased cell survival by the induction of apoptosis, HepG2 cells were cultured with capsaicin with or without SMF exposure and then assessed with flow cytometry. Cells were double stained with Annexin V-FITC and PI. Annexin V has a strong affinity for phosphatidylserine (PS) residues, and therefore can be used as a probe for detecting apoptosis. Fig 4 shows that incubation with 50 μM capsaicin for 72 h did increase the percentage of apoptotic cells from 1.5% in the control group to 32.1%. Co-treatment with SMF exposure significantly enhanced capsaicin-induced HepG2 cell apoptosis to 59.3%.

### Blocking capsaicin-binding TRPV1 affects the synergistic anti-cancer activity of SMF and capsaicin

**Fig 5:**
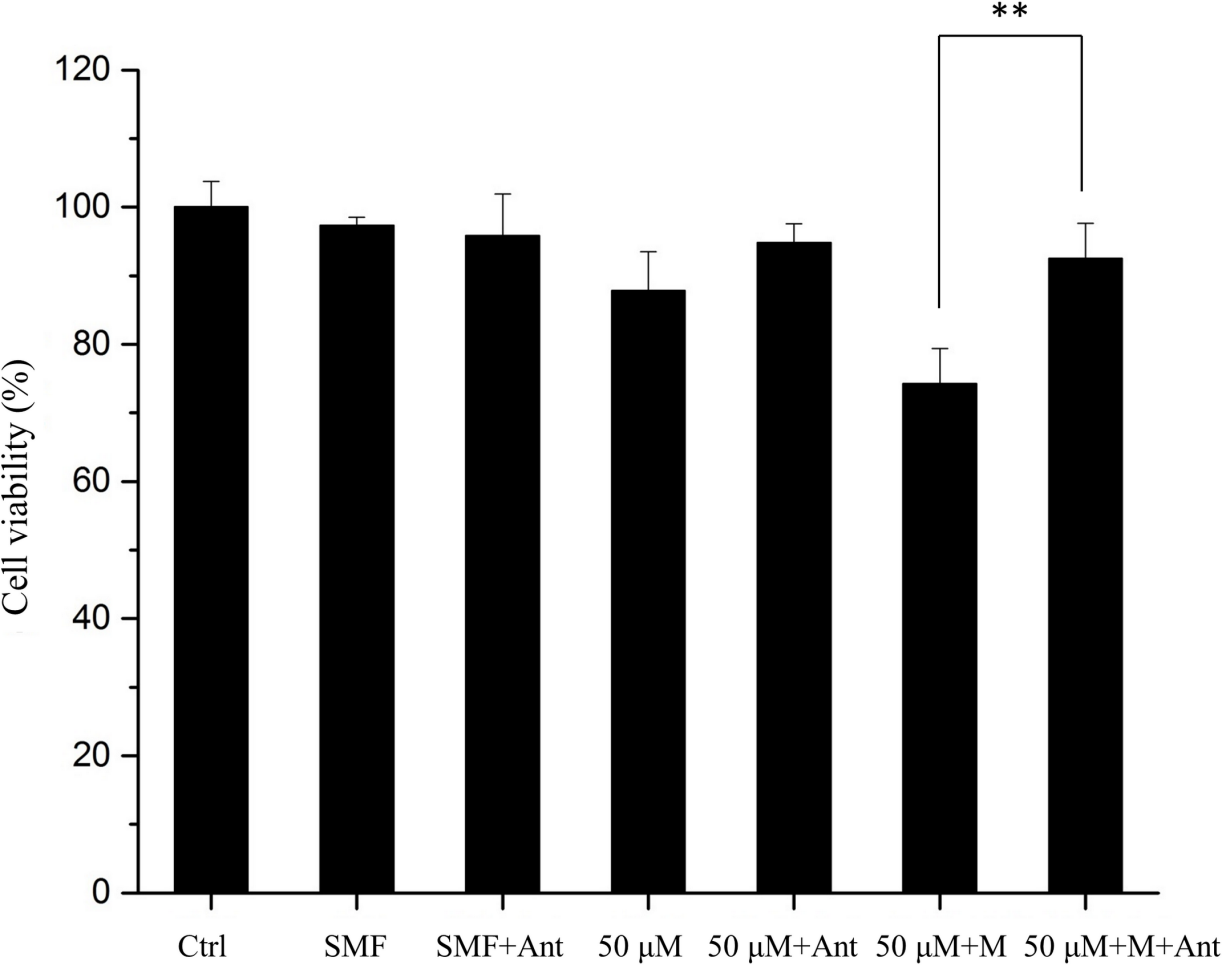
Inhibiting TRPV1 with a function-blocking antagonist negates the effect of SMF enhanced antiproliferative effect. 50 μM: capsaicin, Ant: antagonist (50 nM), M: SMF. Data represent the mean ± SD. **p < 0.01.

In order to realize whether TRPV1 is involved in the synergistic anti-cancer effect, the selective TRPV1 antagonist SB-705498 ((N-(2-bromophenyl)-N’-[((R)-1-(5-tri-fluromethyl-2-pyridyl)pyrrolidin-3-yl)]urea) was incubated with the cells 30 min before SMF and capsaicin treatments [28]. We found that incubation of HepG2 cells with TRPV1 antagonist abrogated the effect of SMF and capsaicin induced cell apoptosis as evidenced by the MTT viability assay (see Fig 5). The result showed that disrupting the function of TRPV1 blocked the enhanced effect of SMF on cancer cell apoptosis. This suggests that the SMF enhanced apoptosis may be mediated by the TRPV1-dependent mechanisms.

### Effects of capsaicin and SMF on intracellular calcium levels

**Fig 6:**
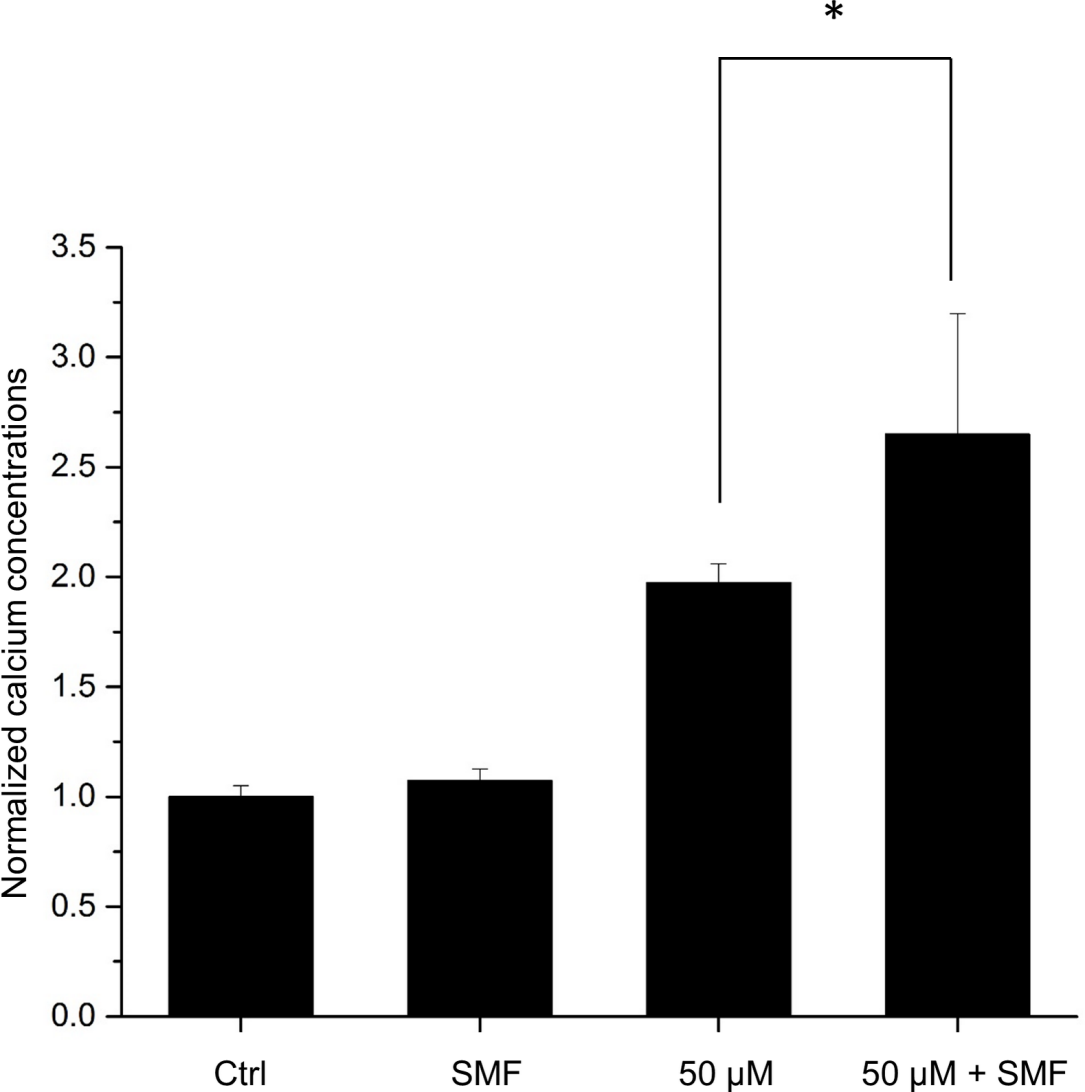
Effects of 50 μM capsaicin and SMF on intracellular calcium levels. Data represent the mean ± SD. *p < 0.05.

The fluorescence of the Rhod-4 loading dye is greatly enhanced once inside the cell and binding to calcium. Intracellular calcium concentrations were measured with a microplate reader at Ex/Em = 532/555 nm. Fig 6 shows that capsaicin treatment greatly increased the intracellular calcium concentration which is a known fact since capsaicin is a TRPV1 channel agonist. On the other hand, SMF alone did not influence the calcium level significantly. Interestingly, the combined treatment of SMF and capsaicin significantly increased the calcium concentration compared with capsaicin only group.

## Discussion

The focus of our research was to investigate the synergistic anti-cancer effect of SMF and capsaicin on HepG2 cells. Liver cancer is one of the most frequent and leading cause of cancer death. The incidence of liver cancer is rising worldwide due to various factors that may cause cirrhosis, including the widely disseminated hepatitis B and C viruses and alcohol use. The therapeutic efficacy of recent surgery and chemotherapy are limited, and some of the chemotherapy drugs may cause more severe side effects in people with cirrhosis [29]. Therefore, research looking for appropriate drugs and method which produce maximum effect and minimum unpleasant side effects is a matter of vital importance. Previous studies have shown that SMF has the ability to enhance the effect of some anti-cancer drugs [14]. However, most studies still remain focusd on conventional chemotherapy drugs. No attempt was made to investigate the combinational effect of SMF and natural compounds. To date, cancer therapy and prevention by natural compounds have shown promising results against various malignancies in vitro and in vivo [30]. Due to their low cytotoxic effects in normal cells, natural products had been a highly useful source for anti-cancer drugs. The novelty of this study is the use of capsaicin, one of the food additive consumed worldwide, in combination with SMF to further inhibit the cancer cells. In agreement with other reports [18], we found capsaicin also induces cell inhibition on HepG2 cells in a dose dependent manner. In this study, we choose lower concentration of capsaicin (< 100 μM) to investigate the biological effects in capsaicin-treated cancer cells under the exposure of SMF. Regarding to the combination of SMF exposure with capsaicin, our results in the cell viability assay have provided clear evidence for an additional synergistic effect of the combination therapy. In comparison to the monotherapy of SMF or capsaicin, the combination treatment yielded a higher inhibition effect in tumor growth. In the present study, we found that 50 μM capsaicin is the most effective parameter to work synergistically with SMF. This result suggests that there is an appropriate treatment level that magnetic field and anti-cancer drugs could inhibit the cancer cell proliferation synergistically.

Literature reports indicate that capsaicin binds to distinct cell surface receptors [31, 32] and can modulate membrane protein function by altering bilayer elasticity [33]. In particular, efforts have been made to understand the specific and selective receptor for capsaicin, TRPV1 [19, 34, 35]. Studies had shown that the activation of TRPV1 is capable of inducing apoptosis and inhibiting cancer cell growth by cell cycle arrest in many different types of cancer, while normal cells remained unharmed [22, 36, 37]. In order to examine whether the capsaicin-induced apoptotic effect was mediated by TRPV1, the TRPV1-specific antagonist SB-705498 [28] was used and incubated with the cells before the capsaicin and SMF treatments. As a result, the enhanced anti-cancer effect was diminished by the TRPV1 blocker. Hence, in this study, the synergistic effect could be due to that SMF increased the binding efficiency of capsaicin for the TRPV1 channel, thereby altering the activation kinetics and the downstream apoptotic pathways. To further confirm that the TRPV1 activity was indeed influenced by SMF and capsaicin, we performed the calcium assay to measure the intracellular calcium levels after the treatments. The results showed that capsaicin indeed activated the TRPV1 channel. Interestingly, while SMF alone did not influence the calcium concentration, SMF and capsaicin synergistically increased the cytoplasmic calcium concentrations. The results confirm the suggestion that TRPV1 activity plays an important role in the SMF enhanced anti-cancer effect. Many studies attempt to provide explanations for the enhancement effect caused by SMF, however, as far as we know, the drug-specific phenomenon could not be explained satisfactorily. Thus, more studies will be necessary to elucidate the role of SMF played on specific anti-cancer drugs. Previous study suggested that during moderate SMF exposure, the reorientation of membrane phospholipids will result in the deformation of imbedded ion channels, and hence influences channel activations [38]. Besides, experimental data had shown that SMF affected the TRPV1-modulated nociceptive processes [39, 40]. Therefore, in this study, part of the mechanism by which SMF affects capsaicin activity may involve a conformational change of TRPV1 ion channel, possibly making it more available to capsaicin. In other words, SMF increases the probability of interaction between capsaicin and the transmembrane ion channel TRPV1 and thus enhances the anti-cancer effect exhibited by capsaicin.

Previous studies have shown that apoptosis is an important procedure for chemotherapeutic agents-induced tumor cell death [41]. In our case, we found that capsaicin induced apoptosis in HepG2 cells, as evidenced by the flow cytometry result. When the capsaicin-treated group was under SMF exposure, the Annexin V positive cells were further increased, indicating the efficacy of capsaicin was indeed enhanced by SMF. It has been postulated that apoptotic signal is regulated by two subgroups of pro-apoptotic proteins and anti-apoptotic proteins [42]. For instance, the balance of the apoptosis-promoting protein Bax and apoptosis-inhibiting protein Bcl-2 subtly controls the apoptotic signaling pathway by regulating mitochondrial function. Bax is a member of the Bcl-2 protein family and its expression is regulated by the tumor suppressor p53 gene [43]. In healthy cells, Bax is located mostly in the cytosol, but upon initiation of apoptotic signaling, it undergoes a conformational change, and becomes more membrane-associated [44]. Basically, it can be realized from earlier studies that Bax increases the mitochondrial membrane permeability by interacting with voltage-dependent ion channel [45] or forming oligomeric pores in mitochondrial outer membrane [46]. This results in the release of cytochrome c and other apoptotic factors, and thus activates caspase-9 which processes the main executioner caspase-3, finally reaching the “point of no return'' in apoptotic pathway [47]. On the contrary, the apoptosis-inhibiting protein Bcl-2 provides survival signals to the cell. Studies have shown that the Bax/Bcl-2 ratio is an important index which determines the susceptibility to apoptosis in cancer cells [25]. In accordance with this, we found that capsaicin increased the Bax/Bcl-2 ratio in HepG2 cells, indicating the apoptotic signal was induced. It was also in agreement with the flow cytometry result showing increased population of apoptotic cells. Moreover, the Bax/Bcl-2 ratio was further increased by SMF exposure, which again revealed the assisted virtue of SMF. In this work, we also showed that the level of active caspase-3 was increased in capsaicin-treated HepG2 cells by immunofluorescence microscopy. Caspase-3 is considered to be the main executioner in the apoptotic process, once it is activated, the apoptotic process is at the point of no return [47]. In the combinational treatment, our results demonstrated for the first time that SMF significantly increased the capsaicin-induced caspase-3 fluorescence intensity, which unambiguously confirms the anti-tumor efficacy of capsaicin can be enhanced by SMF treatment.

In conclusion, we show that capsaicin and SMF could synergistically inhibit HepG2 cancer cell growth through mitochondria-dependent apoptosis pathway. The potency of capsaicin against cancer cells was significantly enhanced by SMF. The underlying mechanism could be due to that SMF induces conformational change of TRPV1 ion channel and facilitates the interaction between capsaicin and TRPV1 channel, thus enhancing the anti-cancer effect exhibited by capsaicin. This study yields important implications for the therapeutic usage of anti-cancer drugs in combination with SMF, which opens new doors in the development of new strategies in fighting cancer with minimum cytotoxicity and side effects. Further studies are needed to understand the specific role SMF played on anti-cancer drugs and to optimize SMF parameters and appropriate drug combinations.

## Supporting information

S1 File Raw data of MTT assay. (RAR)

S2 File Raw data of Western blot analysis. (RAR)

S3 File Raw data of confocal microscope. (ZIP)

S4 File Raw data of flow cytometry. (ZIP)

S5 File Raw data of calcium assay. (RAR)

## References

[1] Markov MS. Magnetic Field Therapy: A Review. Electromagnetic Biology and Medicine. 2007; 26:1–23. doi: 10.1080/15368370600925342 17454079 10.1080/15368370600925342

[2] Jing D, Shen G, Cai J, Li F, Huang J, Wang Y, et al Effects of 180 mT static magnetic fields on diabetic wound healing in rats. Bioelectromagnetics. 2010; 31:640–648. doi: 10.1002/bem.20592 20607739 10.1002/bem.20592

[3] Meng J, Xiao B, Zhang Y, Liu J, Xue H, Lei J, et al Super-paramagnetic responsive nanofibrous scaffolds under static magnetic field enhance osteogenesis for bone repair in vivo. Scientific Reports. 2013; 3:2655 doi: 10.1038/srep02655 24030698 10.1038/srep02655 PMC3772377

[4] Sisken B, Midkiff P, Tweheus A, Markov M. Influence of static magnetic fields on nerve regeneration in vitro. Environmentalist. 2007; 27:477–481.

[5] Ma Q, Chen C, Deng P, Zhu G, Lin M, Zhang L, et al Extremely Low-Frequency Electromagnetic Fields Promote In Vitro Neuronal Differentiation and Neurite Outgrowth of Embryonic Neural Stem Cells via Up-Regulating TRPC1. PLOS ONE. 2016; 11:e0150923 doi: 10.1371/journal.pone.0150923 26950212 10.1371/journal.pone.0150923 PMC4780708

[6] Kanai S, Taniguchi N. Efficacy of static magnetic field for pain of adjuvant arthritis rats. Advances in Bioscience and Biotechnology. 2012; 3:511–515.

[7] Okano H, Ohkubo C. Effects of neck exposure to 5.5 mT static magnetic field on pharmacologically modulated blood pressure in conscious rabbits. Bioelectromagnetics. 2005; 26:469–480. doi: 10.1002/bem.20115 16108042 10.1002/bem.20115

[8] Vergallo C, Dini L, Szamosvölgyi Z, Tenuzzo BA, Carata E, Panzarini E, et al In Vitro Analysis of the Anti-Inflammatory Effect of Inhomogeneous Static Magnetic Field-Exposure on Human Macrophages and Lymphocytes. PLoS ONE. 2013; 8:e72374 doi: 10.1371/journal.pone.0072374 23991101 10.1371/journal.pone.0072374 PMC3753352

[9] Strelczyk D, Eichhorn ME, Luedemann S, Brix G, Dellian M, Berghaus A, et al Static magnetic fields impair angiogenesis and growth of solid tumors in vivo. Cancer biology & therapy. 2009; 8:1756–1762. 19633422 10.4161/cbt.8.18.9294

[10] Tofani S, Cintorino M, Barone D, Berardelli M, De Santi MM, Ferrara A, et al Increased mouse survival, tumor growth inhibition and decreased immunoreactive p53 after exposure to magnetic fields. Bioelectromagnetics. 2002; 23:230–238. 11891753 10.1002/bem.10010

[11] Chionna A, Tenuzzo B, Panzarini E, Dwikat MB, Abbro L, Dini L. Time dependent modifications of Hep G2 cells during exposure to static magnetic fields. Bioelectromagnetics. 2005; 26:275–286. doi: 10.1002/bem.20081 15832333 10.1002/bem.20081

[12] Liu Y, Qi H, Sun R-g, Chen W-f. An investigation into the combined effect of static magnetic fields and different anticancer drugs on K562 cell membranes. Tumori. 2011; 97:386–392. doi: 10.1700/912.10039 21789021 10.1177/030089161109700322

[13] Luo Y, Ji X, Liu J, Li Z, Wang W, Chen W, et al Moderate intensity static magnetic fields affect mitotic spindles and increase the antitumor efficacy of 5-FU and Taxol. Bioelectrochemistry. 2016; 109:31–40. doi: 10.1016/j.bioelechem.2016.01.001 26775206 10.1016/j.bioelechem.2016.01.001

[14] Gellrich D, Becker S, Strieth S. Static magnetic fields increase tumor microvessel leakiness and improve antitumoral efficacy in combination with paclitaxel. Cancer Letters. 2014; 343:107–114. doi: 10.1016/j.canlet.2013.09.021 24075957 10.1016/j.canlet.2013.09.021

[15] Zhang K, Chen W, Bu T, Qi H, Sun R, He X. Decreased P-glycoprotein is associated with the inhibitory effects of static magnetic fields and cisplatin on K562 cells. Bioelectromagnetics. 2014; 35:437–443. doi: 10.1002/bem.21863 25099308 10.1002/bem.21863

[16] Vergallo C, Ahmadi M, Mobasheri H, Dini L. Impact of Inhomogeneous Static Magnetic Field (31.7–232.0 mT) Exposure on Human Neuroblastoma SH-SY5Y Cells during Cisplatin Administration. PLoS ONE. 2014; 9:e113530 doi: 10.1371/journal.pone.0113530 25423171 10.1371/journal.pone.0113530 PMC4244110

[17] Hong Z-F, Zhao W-X, Yin Z-Y, Xie C-R, Xu Y-P, Chi X-Q, et al Capsaicin Enhances the Drug Sensitivity of Cholangiocarcinoma through the Inhibition of Chemotherapeutic-Induced Autophagy. PLoS ONE. 2015; 10:e0121538 doi: 10.1371/journal.pone.0121538 25933112 10.1371/journal.pone.0121538 PMC4416771

[18] Ramos-Torres Á, Bort A, Morell C, Rodríguez-Henche N, Díaz-Laviada I. The pepper's natural ingredient capsaicin induces autophagy blockage in prostate cancer cells. Oncotarget. 2015; 7:1569–1583. 10.18632/oncotarget.6415 PMC4811481 26625315

[19] Darré L, Domene C. Binding of Capsaicin to the TRPV1 Ion Channel. Molecular Pharmaceutics. 2015; 12:4454–4465. doi: 10.1021/acs.molpharmaceut.5b00641 26502196 10.1021/acs.molpharmaceut.5b00641

[20] Holland C, van Drunen C, Denyer J, Smart K, Segboer C, Terreehorst I, et al Inhibition of capsaicin-driven nasal hyper-reactivity by SB-705498, a TRPV1 antagonist. British Journal of Clinical Pharmacology. 2014; 77:777–788. doi: 10.1111/bcp.12219 23909699 10.1111/bcp.12219 PMC4004398

[21] Brito R, Sheth S, Mukherjea D, Rybak L, Ramkumar V. TRPV1: A Potential Drug Target for Treating Various Diseases. Cells. 2014; 3:517–545. doi: 10.3390/cells3020517 24861977 10.3390/cells3020517 PMC4092862

[22] Zheng L, Chen J, Ma Z, Liu W, Yang F, Yang Z, et al Capsaicin enhances anti-proliferation efficacy of pirarubicin via activating TRPV1 and inhibiting PCNA nuclear translocation in 5637 cells. Molecular medicine reports. 2016; 13:881–887. doi: 10.3892/mmr.2015.4623 26648574 10.3892/mmr.2015.4623

[23] Huang S-P, Chen J-C, Wu C-C, Chen C-T, Tang N-Y, Ho Y-T, et al Capsaicin-induced apoptosis in human hepatoma HepG2 cells. Anticancer research. 2009; 29:165–174. 19331147

[24] Chard PS, Bleakman D, Savidge JR, Miller RJ. Capsaicin-induced neurotoxicity in cultured dorsal root ganglion neurons: Involvement of calcium-activated proteases. Neuroscience. 1995; 65:1099–1108. 7617165 10.1016/0306-4522(94)00548-j

[25] Raisova M, Hossini AM, Eberle J, Riebeling C, Orfanos CE, Geilen CC, et al The Bax/Bcl-2 Ratio Determines the Susceptibility of Human Melanoma Cells to CD95/Fas-Mediated Apoptosis. Journal of Investigative Dermatology. 2001; 117:333–340. doi: 10.1046/j.0022-202x.2001.01409.x 11511312 10.1046/j.0022-202x.2001.01409.x

[26] Salakou S, Kardamakis D, Tsamandas AC, Zolota V, Apostolakis E, Tzelepi V, et al Increased Bax/Bcl-2 Ratio Up-regulates Caspase-3 and Increases Apoptosis in the Thymus of Patients with Myasthenia Gravis. In Vivo. 2007; 21:123–132. 17354625

[27] Perlman H, Zhang X, Chen MW, Walsh K, Buttyan R. An elevated bax/bcl-2 ratio corresponds with the onset of prostate epithelial cell apoptosis. Cell death and differentiation. 1999; 6:48–54. doi: 10.1038/sj.cdd.4400453 10200547 10.1038/sj.cdd.4400453

[28] Gunthorpe MJ, Hannan SL, Smart D, Jerman JC, Arpino S, Smith GD, et al Characterization of SB-705498, a potent and selective vanilloid receptor-1 (VR1/TRPV1) antagonist that inhibits the capsaicin-, acid-, and heat-mediated activation of the receptor. Journal of Pharmacology and Experimental Therapeutics. 2007; 321:1183–1192. doi: 10.1124/jpet.106.116657 17392405 10.1124/jpet.106.116657

[29] Pinter M, Trauner M, Peck-Radosavljevic M, Sieghart W. Cancer and liver cirrhosis: implications on prognosis and management. ESMO Open. 2016; 1:e000042 doi: 10.1136/esmoopen-2016-000042 27843598 10.1136/esmoopen-2016-000042 PMC5070280

[30] Manson MM. Cancer prevention–the potential for diet to modulate molecular signalling. Trends in Molecular Medicine. 2003; 9:11–18. 12524205 10.1016/s1471-4914(02)00002-3

[31] Szallasi A, Blumberg PM. Resiniferatoxin and its analogs provide novel insights into the pharmacology of the vanilloid (capsaicin) receptor. Life Sciences. 1990; 47:1399–1408. 2174484 10.1016/0024-3205(90)90518-v

[32] Szallasi A, Cortright DN, Blum CA, Eid SR. The vanilloid receptor TRPV1: 10 years from channel cloning to antagonist proof-of-concept. Nat Rev Drug Discov. 2007; 6:357–372. doi: 10.1038/nrd2280 17464295 10.1038/nrd2280

[33] Lundbæk JA, Birn P, Tape SE, Toombes GES, Søgaard R, Koeppe RE, et al Capsaicin Regulates Voltage-Dependent Sodium Channels by Altering Lipid Bilayer Elasticity. Molecular Pharmacology. 2005; 68:680–689. doi: 10.1124/mol.105.013573 15967874 10.1124/mol.105.013573

[34] Caterina MJ, Schumacher MA, Tominaga M, Rosen TA, Levine JD, Julius D. The capsaicin receptor: a heat-activated ion channel in the pain pathway. Nature. 1997; 389:816–824. doi: 10.1038/39807 9349813 10.1038/39807

[35] Liao M, Cao E, Julius D, Cheng Y. Structure of the TRPV1 ion channel determined by electron cryo-microscopy. Nature. 2013; 504:107–112. doi: 10.1038/nature12822 24305160 10.1038/nature12822 PMC4078027

[36] Amantini C, Mosca M, Nabissi M, Lucciarini R, Caprodossi S, Arcella A, et al Capsaicin-induced apoptosis of glioma cells is mediated by TRPV1 vanilloid receptor and requires p38 MAPK activation. Journal of Neurochemistry. 2007; 102:977–990. doi: 10.1111/j.1471-4159.2007.04582.x 17442041 10.1111/j.1471-4159.2007.04582.x

[37] CLARK R, LEE S-H. Anticancer Properties of Capsaicin Against Human Cancer. Anticancer Research. 2016; 36:837–843. 26976969

[38] Rosen AD. Mechanism of action of moderate-intensity static magnetic fields on biological systems. Cell Biochemistry and Biophysics. 2003; 39:163–173. doi: 10.1385/CBB:39:2:163 14515021 10.1385/CBB:39:2:163

[39] Sándor K, Helyes Z, Gyires K, Szolcsányi J, László J. Static magnetic field-induced anti-nociceptive effect and the involvement of capsaicin-sensitive sensory nerves in this mechanism. Life Sciences. 2007; 81:97–102. doi: 10.1016/j.lfs.2007.04.029 17568617 10.1016/j.lfs.2007.04.029

[40] Del Seppia C, Mezzasalma L, Choleris E, Luschi P, Ghione S. Effects of magnetic field exposure on open field behaviour and nociceptive responses in mice. Behavioural Brain Research. 2003; 144:1–9. 12946589 10.1016/s0166-4328(03)00042-1

[41] Fang EF, Zhang CZY, Fong WP, Ng TB. RNase MC2: a new Momordica charantia ribonuclease that induces apoptosis in breast cancer cells associated with activation of MAPKs and induction of caspase pathways. Apoptosis. 2011; 17:377–387. 10.1007/s10495-011-0684-z 22134530

[42] Kelly PN, Strasser A. The role of Bcl-2 and its pro-survival relatives in tumourigenesis and cancer therapy. Cell Death and Differentiation. 2011; 18:1414–1424. doi: 10.1038/cdd.2011.17 21415859 10.1038/cdd.2011.17 PMC3149740

[43] Knudson CM, Johnson GM, Lin Y, Korsmeyer SJ. Bax Accelerates Tumorigenesis in p53-deficient Mice. Cancer Research. 2001; 61:659–665. 11212265

[44] Hsu Y-T, Wolter KG, Youle RJ. Cytosol-to-membrane redistribution of Bax and Bcl-XL during apoptosis. Proceedings of the National Academy of Sciences. 1997; 94:3668–3672. 10.1073/pnas.94.8.3668 PMC20498 9108035

[45] Shi Y, Chen J, Weng C, Chen R, Zheng Y, Chen Q, et al Identification of the protein–protein contact site and interaction mode of human VDAC1 with Bcl-2 family proteins. Biochemical and Biophysical Research Communications. 2003; 305:989–996. 12767928 10.1016/s0006-291x(03)00871-4

[46] Dejean LM, Martinez-Caballero S, Manon S, Kinnally KW. Regulation of the mitochondrial apoptosis-induced channel, MAC, by BCL-2 family proteins. Biochimica et Biophysica Acta (BBA)—Molecular Basis of Disease. 2006; 1762:191–201. 16055309 10.1016/j.bbadis.2005.07.002

[47] Enari M, Talanian RV, Wrong WW, Nagata S. Sequential activation of ICE-like and CPP32-like proteases during Fas-mediated apoptosis. Nature. 1996; 380:723–726. doi: 10.1038/380723a0 8614469 10.1038/380723a0

