# Supplementary figures and images for: Static magnetic field enhances the anticancer efficacy of capsaicin on HepG2 cells via capsaicin receptor TRPV1
Source: PLoS One. 2018 Jan 16;13(1):e0191078. doi: 10.1371/journal.pone.0191078 (PMC5770067; doi:10.1371/journal.pone.0191078)

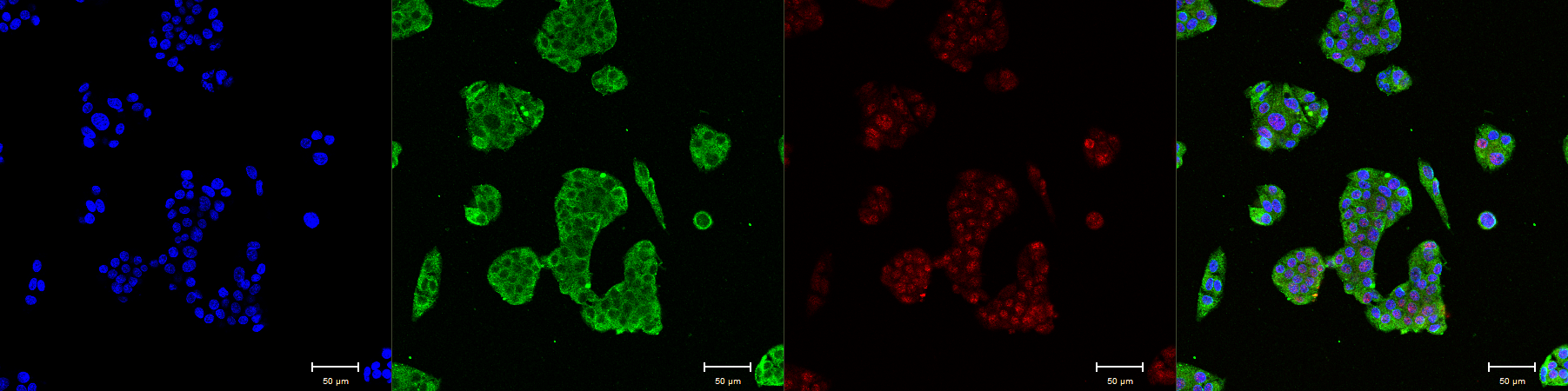

Supplement: S3 File — (ZIP) [file pone.0191078.s003.zip › S3_confocal/50uM.tif]

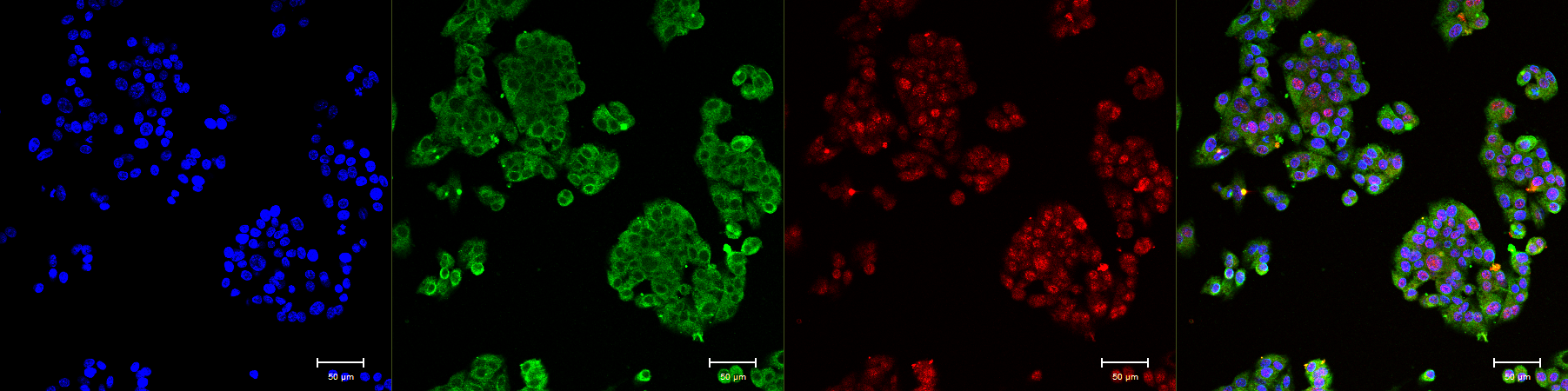

Supplement: S3 File — (ZIP) [file pone.0191078.s003.zip › S3_confocal/50uM+SMF.tif]

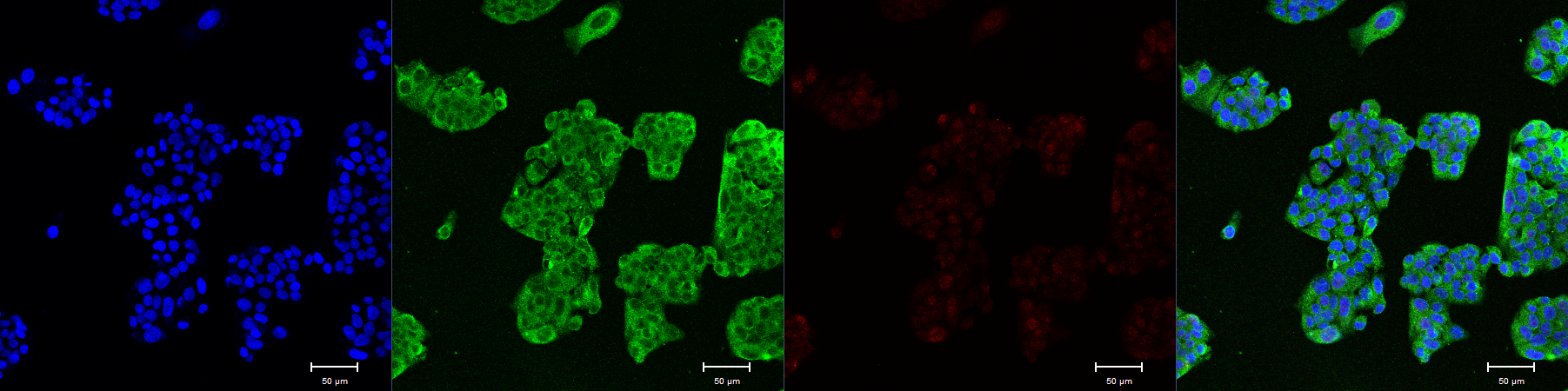

Supplement: S3 File — (ZIP) [file pone.0191078.s003.zip › S3_confocal/CTRL.tif]
