# Supplementary material for: Static magnetic field enhances the anticancer efficacy of capsaicin on HepG2 cells via capsaicin receptor TRPV1
Source: PLoS One. 2018 Jan 16;13(1):e0191078. doi: 10.1371/journal.pone.0191078 (PMC5770067; doi:10.1371/journal.pone.0191078)
Supplement: S4 File — (ZIP) [file pone.0191078.s004.zip › S4_flow.pdf]

FACSDiva Version 6.1.3

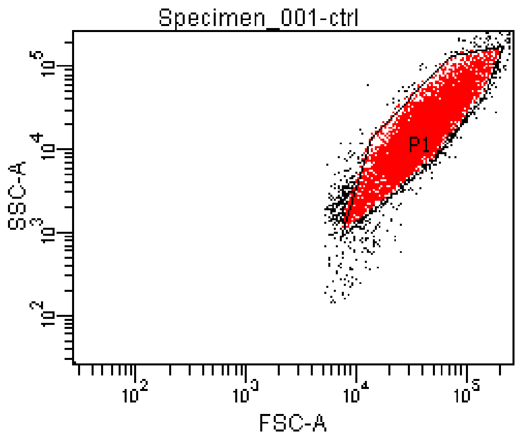

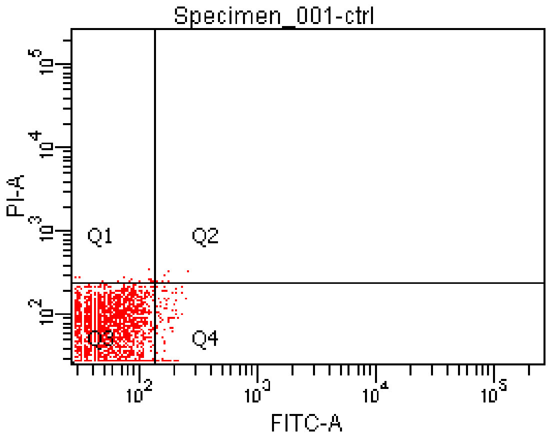

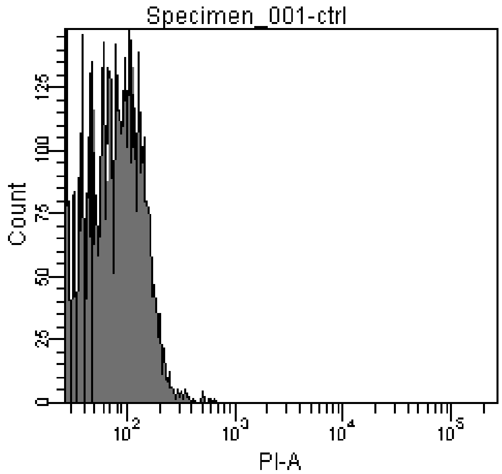

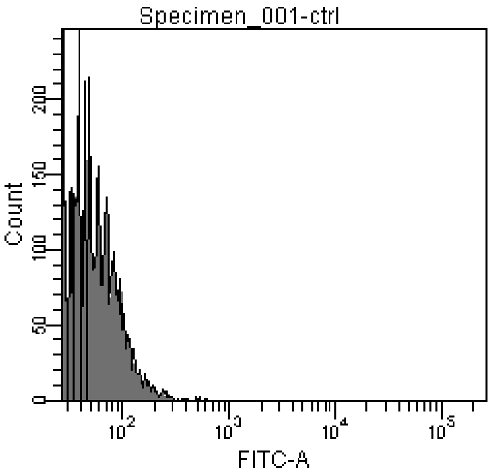

Tube: ctrl

Population	#Events	%Parent	%Total
All Events	10,650	####	100.0
P1	9,905	93.0	93.0
Q1	17	0.2	0.2
Q2	31	0.3	0.3
Q3	10,439	98.0	98.0
Q4	163	1.5	1.5

FACSDiva Version 6.1.3

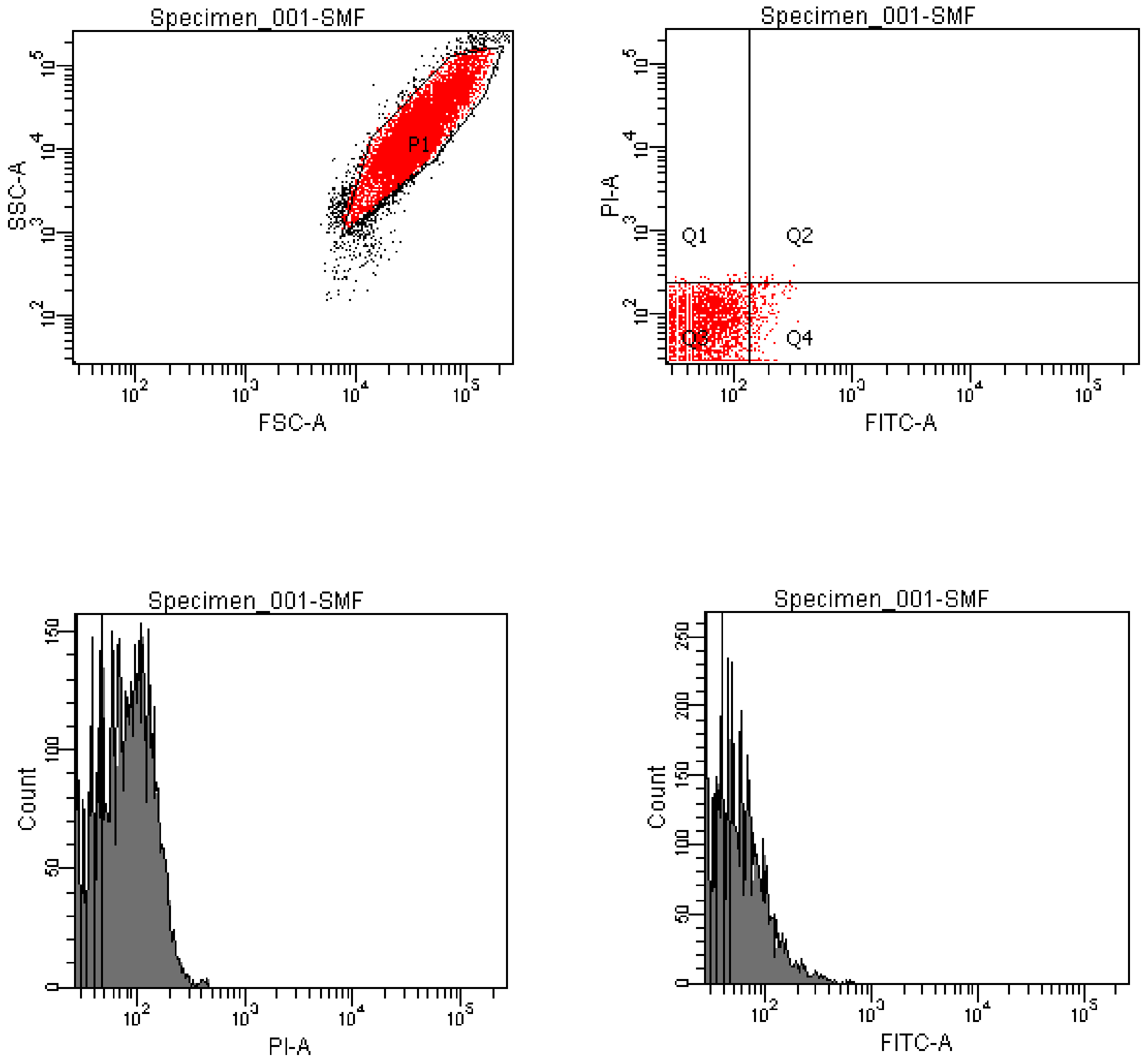

Tube: SMF

Population	#Events	%Parent	%Total
All Events	10,888	####	100.0
P1	9,963	91.5	91.5
Q1	17	0.2	0.2
Q2	42	0.4	0.4
Q3	10,500	96.4	96.4
Q4	329	3.0	3.0

FACSDiva Version 6.1.3

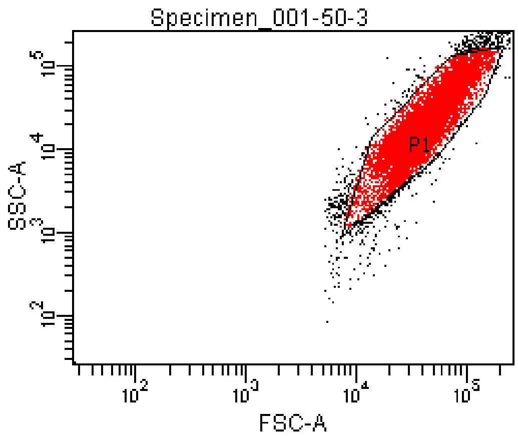

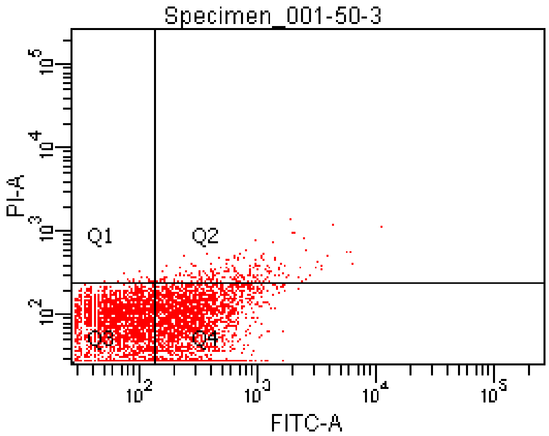

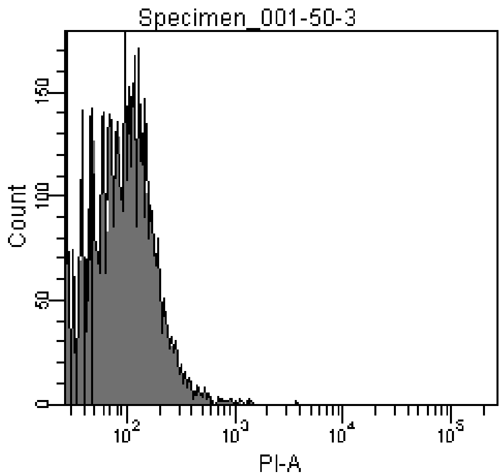

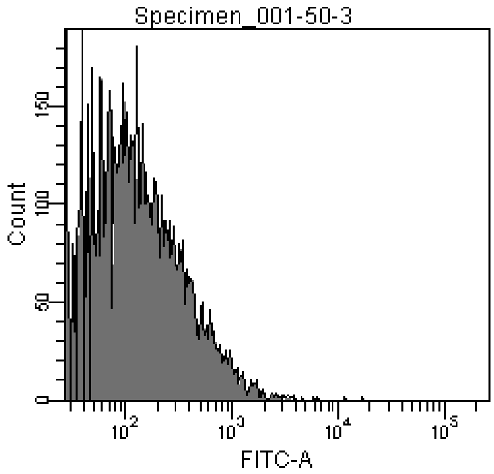

Tube: 50-3

Population	#Events	%Parent	%Total
All Events	10,791	####	100.0
P1	9,973	92.4	92.4
Q1	8	0.1	0.1
Q2	303	2.8	2.8
Q3	7,011	65.0	65.0
Q4	3,469	32.1	32.1

FACSDiva Version 6.1.3

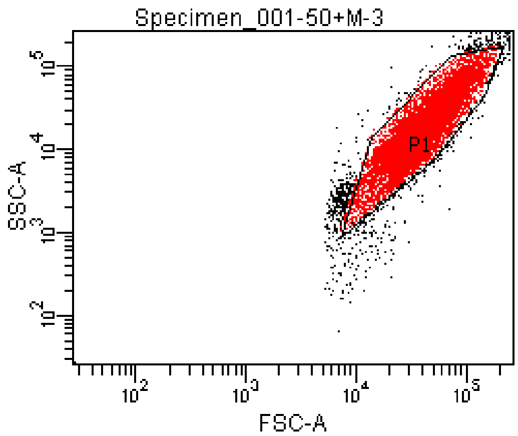

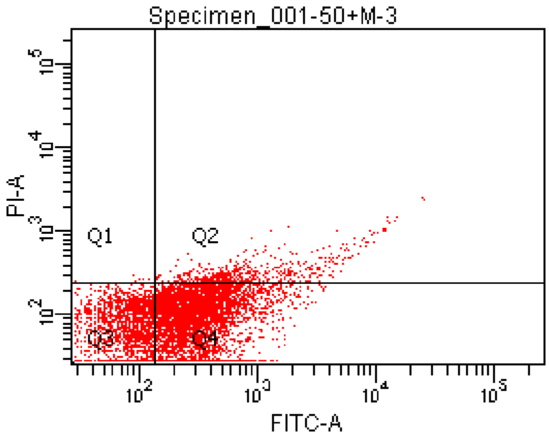

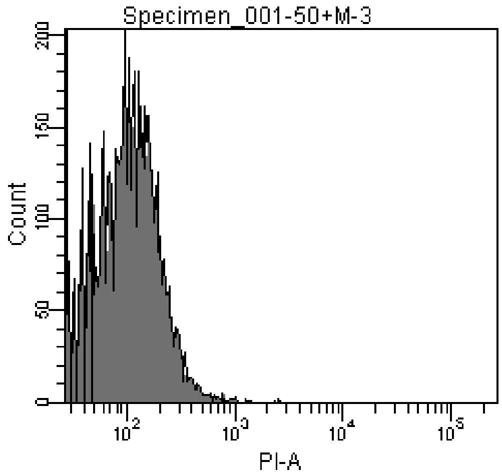

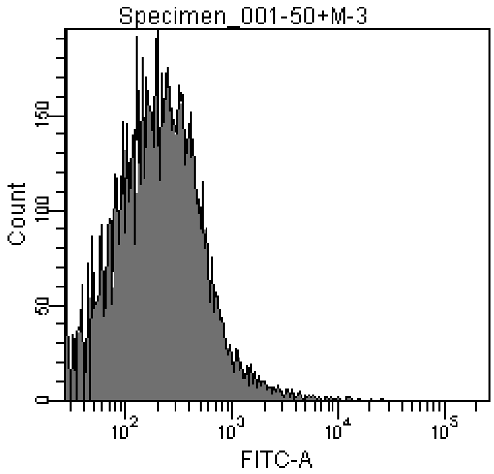

Tube: 50+M-3

Population	#Events	%Parent	%Total
All Events	10,918	####	100.0
P1	10,105	92.6	92.6
Q1	4	0.0	0.0
Q2	473	4.3	4.3
Q3	3,972	36.4	36.4
Q4	6,469	59.3	59.3
